# The Antibiotic Resistome and Its Association with Bacterial Communities in Raw Camel Milk from Altay Xinjiang

**DOI:** 10.3390/foods12213928

**Published:** 2023-10-26

**Authors:** Yanan Qin, Wanting Huang, Jie Yang, Yan Zhao, Min Zhao, Haotian Xu, Minwei Zhang

**Affiliations:** Xinjiang Key Laboratory of Biological Resources and Genetic Engineering, College of Life Science & Technology, Urumqi 830046, China; huangwt0429@sina.com (W.H.); yangjie234@xju.edu.cn (J.Y.); zhaoyan980818@sina.com (Y.Z.); zhaoinmchen@sina.com (M.Z.); xuhaotian0202@163.com (H.X.); zhang78089680@sina.com (M.Z.)

**Keywords:** antibiotic resistance genes, high–throughput qPCR, bacteria community, raw camel milk, network analysis

## Abstract

Raw camel milk is generally contaminated with varied microbiota, including antibiotic–resistant bacteria (ARB), that can act as a potential pathway for the spread of antibiotic resistance genes (ARGs). In this study, high–throughput quantitative PCR and 16S rRNA gene–based Illumine sequencing data were used to establish a comprehensive understanding of the antibiotic resistome and its relationship with the bacterial community in Bactrian camel milk from Xinjiang. A total of 136 ARGs and up to 1.33 × 10^8^ total ARG copies per gram were identified, which predominantly encode resistance to β–lactamas and multidrugs. The ARGs’ profiles were mainly explained by interactions between the bacteria community and physicochemical indicators (77.9%). Network analysis suggested that most ARGs exhibited co–occurrence with *Corynebacterium*, *Leuconostoc* and MGEs. Overall, raw camel milk serves as a reservoir for ARGs, which may aggravate the spread of ARGs through vertical and horizontal gene transfer in the food chain.

## 1. Introduction

Bactrian camels, which belong to *C. bactrianus,* are mainly found in Central Asia, Mongolia and China [1]. Xinjiang, the northwest province of China, has a long history of *C. bactrianus* domestication for husbandry as one of the primary corridors of the ancient Silk Road [2]. Once, Bactrian camels were used for transport by local herders. However, currently this animal represents one of the most valuable livestock resources in Xinjiang due to their various byproducts, such as lambsdown, camel meat and camel milk [3]. Bactrian camel milk has, similarly to donkey milk [4], multiple pharmacological effects, such as antifatigue and anti–inflammatory properties, and it is used for the adjuvant treatment of diabetes and to inhibit the growth of tumors [5]. Due to these factors, the demand for raw camel milk is on the rise. Despite the nutrients and reported health benefits of camel milk, raw milk is contaminated with varied microbiota including foodborne pathogens (*Pseudomonas*, *Streptococcus*, *Acinetobacter*) and probiotics (*Lactobacillus*, *Lactococcus*) [6].

Antibiotics are commonly used in dairy farming for the treatment of a number of prevalent diseases. The continued use of antibiotics leads to antibiotic resistance in potential pathogenic bacteria in camel milk [7]. For example, methicillin–resistant *Staphylococcus aureus* (MRSA) strains isolated from pasteurized camel milk were resistant to eight antibiotics, which included streptomycin, colistin, polymyxin B and so on [8]. Furthermore, antibiotic resistance genes (ARGs) are the genetic basis of antibiotic resistance bacteria (ARB). The presence of ARGs is also the root cause of bacterial resistance, for example, *Pseudomonas fluorescens* isolated from raw milk showed phenotypic resistance to ampicillin and clavulanate due to the containment of β–lactamas class resistance genes in the genome [9]. Hence, ARB and ARGs, which are widespread in livestock environments and foodstuffs, were shown to be more harmful than antibiotics [10]. On the one hand, ARGs on ARB genomes are inherited by the next generation via vertical gene transfer (VGT). Simultaneously, the horizontal gene transfer (HGT) of ARGs through mobile genetic elements (MGEs) among bacteria, which may lead to the dissemination of the resistant bacterial populations [11], is more serious. In particular, HGT has led to the evolution of resistant foodborne pathogens and probiotics, such as multidrug–resistant *Pseudomonas* strains, kanamycin–resistant *Serratia marcescens* and so on, from bulk tank milk [12]. A recent study reported that abundant aminoglycoside resistance genes have been detected in retail raw milk and dairy products, suggesting that cow milk has been enriched with ARGs [13]. Not all ARGs are destroyed in the pasteurization process; they can remain active and are capable of spreading to *Staphylococcus aureus* during the storage period [14]. Thus, incorporating camel milk into a daily diet might promote the spread of ARGs to the food chain and human gastrointestinal tract. However, it appears that there is a lack of a complete familiarization with the antibiotic resistome in raw camel milk. In addition, several studies have documented raw camel milk microbiota, but they have mostly focused on Bactrian camels in Inner Mongolia [6,15]. Nevertheless, thorough surveys, utilizing high–throughput sequencing to examine the microbiota and resistome content of raw camel milk in Xinjiang, remain limited.

Hence, the aim of this study was to analyze ARGs in camel milk samples from Xinjiang camel farms and the investigation of the antibiotic resistome and its association with the bacterial community by performing high–throughput profiling. For this purpose, the prevalence and variety of ARGs and mobile genetic elements (MGEs) in raw camel milk samples collected from four camel farms in Xinjiang were characterized using high–throughput quantitative PCR (HT–qPCR) chip technology. The ARG profiles were subsequently associated with bacterial community structures in the camel milk samples and assessed via 16S rRNA amplicon sequencing using variation partitioning analysis (VPA), redundancy analysis (RDA) and Procrustes analysis. Moreover, VPA explored the effect of physicochemical factors, MGEs, and the bacterial community on ARG variation. Network analysis was further employed to identify the bacterial hosts of ARGs and the mode of transmission of ARGs in raw Bactrian camel milk.

## 2. Materials and Methods

### 2.1. Sample Collection

A total of 12 milk samples, representative of four groups with triplicated Bactrian camel milk fed on the same feed type, were obtained from four middle–scale camel farms (population ≥ 250 for each farm) in August 2021. The four middle–scale camel farms with the same captive mode were located in Fuhai (C1, C2, C3 farms; 87°00′ to 89°04′ E, 45°00′ to 48°10′ N) and Jeminai (C4 farm; 85°33′ to 87°09′ E, 47°00′ to 47°59′ N), Altay Xinjiang. In detail, for each farm, Bactrian camels were milked twice per day and the milk was transferred to a milk storage tank. We sampled the raw camel milk from the tank on two consecutive days at 6 a.m., which was then mixed and divided into three biological replicates. All 12 raw Bactrian camel milk samples were encapsulated in single 200 mL sterile plastic sample pouches, immediately placed on dry ice, transported to the laboratory and stored at −20 °C until subsequent analysis. The physicochemical parameters of the camel milk were analyzed using a milk composition analyzer (FOSS, Hillerod, Denmark).

### 2.2. 16S rRNA Gene Amplication, Sequencing and Data Processing

DNA was extracted using a FastDNA SPIN Kit for soil (MPbio, Santa Ana, CA, USA) according to the manufacturer’s recommendations. We used spectrophotometer ND–8000 (Thermo Fisher Scientific, Waltham, MA, USA) and 1.5% agar gel electrophoresis to check the quality and concentration of DNA. The V3–V4 variable regions of the bacterial 16S rRNA gene were amplified, purified, quantified, pooled and sequenced on an Illumina NovaSeq6000 platform (BIOTREE, Shanghai, China). The raw paired–end reads of the filtered adaptor, low–quality reads and chimera sequences were assembled to generate effective tags. At 97% similarity, the Uparse algorithm (Uparse version 7.0.1001) was used to define OTUs [16]. The highest frequency sequence as a representative sequence of each OTU was selected and assigned to a taxonomy using the Mothur method (version 1.8.0) and SSUrRNA database (SILVA138) with a confidence threshold of 0.80–1 (80–100%) [17]. MUSCLE aligne (version 3.8.31) was used for sequencing, and Qiime (version 1.9.1) was employed to determine alpha and beta diversity. Alpha diversity levels, which were estimated using Chao1, Shannon, Simpson and ACE, and the bacterial OTU diversity level were compared by generating rarefaction curves. The Bray–Curtis distances and unweighted and weighted Unifrac metrics, followed by the Tukey test and principal coordinate analysis (PCoA), were used to compare the differences in bacteria communities.

### 2.3. High–Throughput Quantitative PCR (HT–qPCR) and Data Processing

The abundance of ARGs in the camel milk samples was evaluated via high–throughput quantitative PCR (HT–qPCR) using the SmartChip Real–time PCR System (Xuanchen Biotechnology Co., Ltd., Xi’an, China), as previously described [18]. A total of 344 genes (Table S1) were used, consisting of 316 ARGs that conferred resistance to the main antibiotic classes, 27 MGEs, and 1 16S rRNA gene. All qPCRs ran two chips in total and were conducted with one technical duplication. The qPCR results were analyzed automatically using the instrument’s qPCR software (version 2.7.0.1). Only the curve–fitting analysis was satisfied before it was regarded as positive and used in further analysis. For the definition of the detection limit for single PCR reactions, a threshold cycle (CT) of 35 was chosen. The formula as described previously, with a slight modification, was used to calculate the relative copy numbers of MGEs and ARGs (Equation (1)) [19]:

relative gene copy numbers = 10^(35−C^_T_^)/(10/3)^. (1)

After, quantifications of the 16S rRNA gene were transformed to absolute copy numbers (Equation (2)):

absolute copy number = absolute copy number of 16S rRNA (relative copy number/relative copy number of 16S rRNA).(2)

The normalized gene copy numbers were computed as follows (Equation (3)):

normalized copy numbers = relative copy number/4 × relative 16S rRNA copy numbers.(3)

The relative abundance of ARGs was computed as the ratio of each ARG’s relative copy number to the sample’s corresponding 16S rRNA gene copy number [19].

### 2.4. Statistical Analysis

To compute averages, standard deviation and standard error, Microsoft Excel 2021 (Microsoft, Redmond, WA, USA) was applied. Alpha diversity values (Chao1, Simpson, ACE and Shannon indexes) and unweighted UniFrac and Bray–Curtis distances were calculated using Qiime software version 1.9.1 (QIIME development team, Aurora, CO, USA). Beta diversity was used to measure the complexity of species between samples. Rarefaction curves, PCoA, the Tukey test, the *t*–test and the Mantel test were performed using R software v 2.15.3 (R Core Team, Wien, Austria). Canoco 5.0 software was used for principal component analysis (PCA, Euclidean distance-based). Procrustes analysis for correlation analysis between ARGs and bacterial communities was performed on the Tutools platform. Redundancy analysis (RDA) of the relationships among bacterial communities, ARGs, physicochemical factors and MGEs was performed using Canoco 5.0 software. The Majorbio Cloud Platform online tool was used to analyze variation partitioning analysis (VPA). Spearman correlation analysis was employed to determine the associations of these ARGs with the bacterial phylum level. Network analysis (OmicStudio tools) was performed to investigate the co–occurrence patterns of ARGs with bacterial phylum according to Spearman’s rank correlations. Origin 2021 (OriginLab, Northampton, MA, USA) was used for the construction of bar charts.

## 3. Results and Discussion

### 3.1. Physicochemical Parameters of Camel Milk

The amounts of protein, fat, nonfat milk solids and lactose in Xinjiang Bactrian camel milk were detected to range from 3.7–3.8%, 4.1–5.2%, 9.4–9.6%, 4.98–5.09% and 0.7–0.72%, respectively (Table 1). These parameters were consistent with the ash, lactose, and protein contents that have been determined for Bactrian camel milk in Inner Mongolia and Mongolia [6,20]. However, the fat content found here was approximately 0.45% higher than previously reported for Bactrian camel milk [20].

### 3.2. The Bacterial Community Structure of Camel Milk

**Figure 1:**
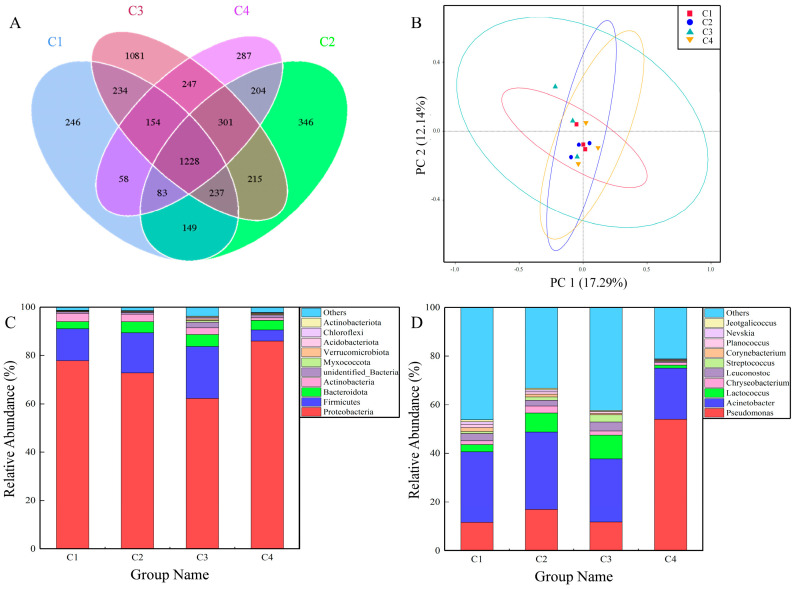
Bacterial communities structures in camel milk from four farms: (**A**) OTUs distribution (Venn diagram) among 16S rRNA transcript amplicons; (**B**) principal coordinate analysis (PCoA) based on Unifrac unweighted distance; the top 10 species in each grouping in terms of maximum abundance at the (**C**) phylum and (**D**) genus levels.

A total of 298,638 sequences were generated from all 16 camel milk samples after quality filtering and assembling, with sequences per camel milk sample ranging from 69,224 to 79,068. The Shannon, Simpson, Chao1 and ACE indexes showed similar species diversity and richness among groups of 4.26–5.59, 0.79–0.9, 1260.25–1595.06, 1352.62–1657.44 and 1352.62–1657.44, respectively (Table S2). The Shannon and Simpson indexes of raw camel milk in Kuwait were 4.12 and 0.94, approximately the same as Bactrian camel milk here [21]. The rarefaction curves indicated that the sequencing depth was sufficient and the highest species diversity was recorded for C3 (Figure S1). Moreover, similar results were obtained when clustering OTUs at the 97% similarity level. The number of unique OTUs in the four farms were 246, 346, 1081 and 287, as shown in the Venn diagram (Figure 1A). The number of shared OTUs in the camel milk from the four farms was 1228, which accounted for approximately 50% of the OTUs from each farm (Figure 1A).

Principal coordinate analysis (PCoA) and principal component analysis (PCA) showed that the repeated samples from each farm had similar clustering (Figure 1B and Figure S2). The bacterial communities from different farms (C1, C2, C3 and C4) were also similar to a certain degree. However, the composition of the bacterial communities at the phylum and genus level in different camel milk samples displayed variation (Figure 1C,D). Proteobacteria, Firmicutes, Actinobacteria and Bacteroidota were the four most dominant phyla, representing more than 90% of the total bacterial taxa (Figure 1C). The major phyla in previous studies of Bactrian camel milk, raw cow milk and goat milk were Proteobacteria and Firmicutes, which is consistent with this paper [15,22,23]. At the level of genus, the dominant genus was *Acinetobacter*, with relative abundances up to 29%, 32% and 26% in the C1, C2 and C3 samples, respectively. However, compared with C1, C2 and C3, the dominant genus of the C4 sample was *Pseudomonas* with relative abundances up to 54% (Figure 1D). *Lactococcus* and *Leuconostoc* were detected in the camel milk samples at levels of 6%, 106%, 14% and 2%, respectively, which are fermentor strains that produce flavor during milk fermentation and improve the quality of dairy products [24,25]. *Chryseobacterium* was also found in all camel milk samples with relative abundances of 1.58%, 2.86%, 1.67% and 1.14%, respectively (Figure 1D). This is a Gram–negative bacterium and food spoilage bacterium usually found in soil, water, and plants [26]. In a previous study, *Lactococcus*, *Acinetobacter* and *Pseudomonas* species accounted for >62% of the 2906 bacterial strains identified in raw milk samples [27]. Similar to the results of the current study, *Pseudomonas* spp., *Acinetobacter* spp. and *Lactococcus* spp. accounted for 44%, 57%, 48% and 76% in all camel milk samples. Additional, *Pseudomonas* species have been classified as being at high risk for antibiotic resistance as they can sustain viability in the aquatic environment for extended periods and raise the risk of transmitting ARGs and MGEs [12,28].

### 3.3. Antibiotic Resistant Genes in Camel Milk

**Figure 2:**
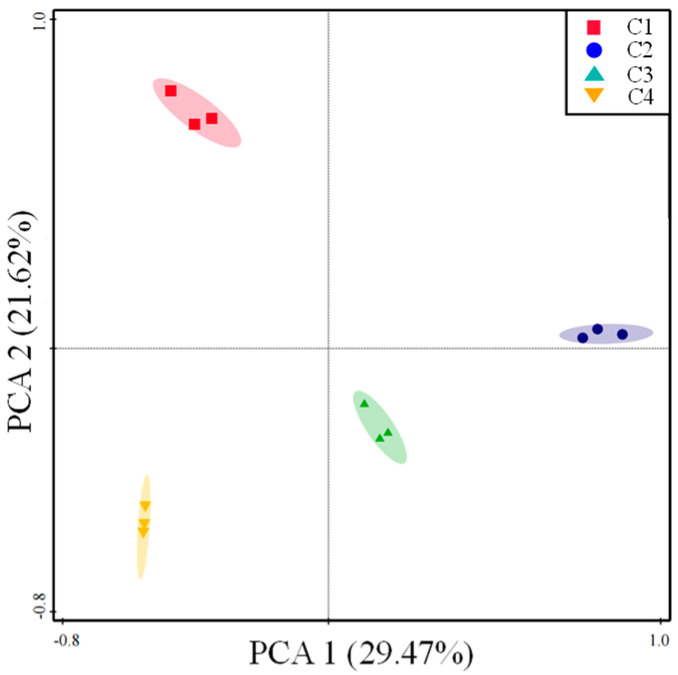
The overall distribution pattern of ARGs in camel milk from four farms is represented here using Euclidean distance-based principal component analysis (PCA). ARG data are represented on a HT–qPCR basis.

**Figure 3:**
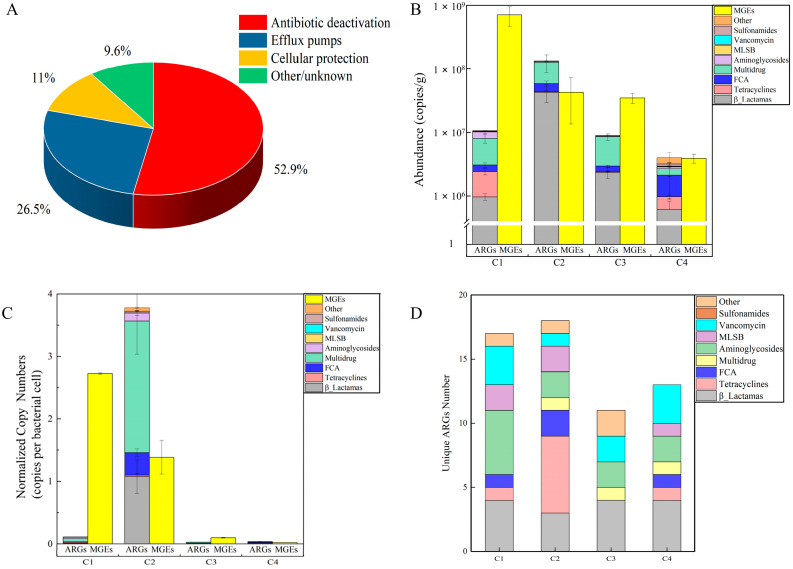
ARGs identified in camel milk from four farms. (**A**) ARGs were classified based on mechanism of resistance. (**B**) The sum of ARG and MGE copy numbers that conferred resistance to a specific antibiotic class was the absolute copy number. (**C**) The number of ARGs per bacterial cell expressed as a normalized copy number of ARGs. (**D**) Number of unique ARGs in camel milk. FCA: quinolone, florfenicol, fluoroquinolone, amphenicol and chloramphenicol resistance genes. MLSB: macrolide–lincosamide–streptogramin B resistance genes.

The distribution of ARGs in camel milk samples was revealed via PCA. Euclidean distance–based PCA analysis clearly demonstrated that camel milk resistome profiles were grouped by farm (Figure 2). The first two PCAs explained 51.09% of the ARGs’ variance, where C1 and C2 were separated from C4 along PCA2 and PCA1, which explained the 21.62% and 29.47% variations, suggesting that variance in the ARGs’ profiles occurred between the farms. A total of 118 ARG subtypes and MGEs were identified in the dairy farms [29]. However, a total of 153 MGEs and ARGs were detected in all camel milk samples that conferred resistance to a wide variety of antibiotics. All major resistance mechanisms, including antibiotic inactivation at 52.9%, efflux pumps at 26.5% and cellular protection at 11%, were represented in the resistance genes (Figure 3A).

Different camel farms have different abundances and varieties in the distributions of their ARGs. The number of ARGs and MGEs detected in C2 (78) was higher than in the others (C1, C3 and C4, approximately 65) (Figure S3). ARGs with resistance to β–lactamas, multidrugs, quinolone, fluoroquinolone, florfenicol, amphenicol, and chloramphenicol (FCA) were the most prevalent resistance genes, while the highest copy number of MGEs detected was in C1 (Figure 3B). ARGs and MGEs detected in the camel milk were very high in terms of abundance, with a range of 4.01 × 10^6^ to 1.33 × 10^8^ and 3.91 × 10^6^ to 7.08 × 10^8^ copies per gram (Figure 3B). The normalized copy numbers of ARGs and MGEs were approximately 3.78 and 2.72, respectively, which were the maximum values in C2 and C1 (Figure 3C). The 30 ARGs shared by the four investigated camel milks mainly conferred resistance to tetracyclines, sulfonamides and β–lactamas (Figure S4). Consistent with the results of our study, tetA, tetB, sul1 and sul2 have previously been proposed to be the most prevalent and abundant ARGs in cow cheese [30]. Unique ARGs per farm were also identified, with a maximum number of 18 unique ARGs in C2, which consisted of aminoglycosides and FCA resistance genes (Figure 3D). Compared to the quantity of ARGs detected in retail raw milk, the present study detected approximately 88 additional ARGs [13]. Overall, the camel milk samples showed widespread ARG profiles. Mastitis is a prevalent disease for camels [31] that is generally treated by injection or topical application of antibiotics, such as penicillin, streptomycin, and oxytetracycline [32]. However, the extensive use of antimicrobial agents on animals used for food products exerts a potent selective pressure that increases resistance between bacteria. Even subinhibitory levels of antibiotics have been shown to trigger the differential expression of ARGs in several studies [33,34]. In addition, adding low doses of antibiotics to feed additives for prophylactic purposes also increases the level of ARGs [35]. So, the high level of ARGs in camel milk indicates that β–lactamas, tetracyclines and aminoglycosides are widely used in camel farms.

### 3.4. Relationship between Physicochemical Indicators, ARGs and Bacterial Community Composition

The total number of ARGs of absolute abundance in the camel milk was strongly associated with the total fat concentration in the camel milk (Pearson correlation coefficient = 0.823, *p* < 0.01) (Table S3). Meanwhile, bacterial abundance was significantly related to the abundance of ARGs (Pearson correlation coefficient = 0.699, *p* < 0.05), with ARGs endowing aminoglycoside, FCA, multidrug and β–lactamas resistance (Table S4). Furthermore, the total number of MGEs and transposons of absolute abundance were extremely significantly related to the ARGs that confer resistance to the remaining classes of antibiotics (*p* < 0.001) (Table S4).

**Figure 4:**
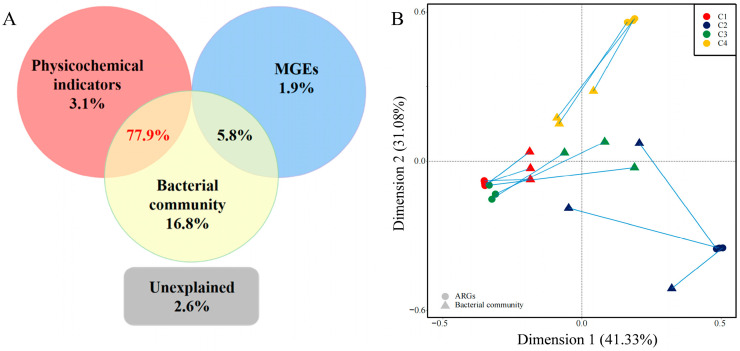
(**A**) Variation partitioning analysis (VPA) analyzes the relationship among physicochemical indicators, bacterial communities, MGEs (mobile genetic elements) and ARGs. (**B**) Procrustes analysis showing the correlation between bacterial community structure and ARGs. Circular dots represent ARG profiles and triangular dots represent bacterial communities, with each farm shown in a different color.

Variation partitioning analysis (VPA) was used to analyze the influence of physicochemical factors, MGEs and bacterial communities on ARG variation (Figure 4A). Bacterial communities revealed the variation (16.8%), which was much higher than that illustrated by physicochemical indicators (3.1%) and MGEs (1.9%) (Figure 4A). Interactions between bacterial communities and MGEs accounted for 5.8% of the variation. The interactions between bacterial communities and physicochemical indicators contributed to a considerably larger variation (77.9%), indicating that they were the main factors affecting the variation of ARGs in the camel milk samples (Figure 4A). Only 2.6% of the ARG variation was unexplained, suggesting that the selected indicators could effectively explain the ARG variation in camel milk samples. Previous researchers found that environmental factors and bacterial communities played a dominant role in shaping ARGs [35,36], for example, the spontaneous fermentation process of milk at room temperature shapes the ARG spectrum by changing physicochemical properties and bacterial communities [13]. The correlation between MGEs and MLSB, tetracyclines and vancomycin resistance genes were found in this study (*p* < 0.001). Moreover, redundancy analysis (RDA) was employed for analyzing the correlations among physicochemical indicators, ARGs and bacterial communities (Figure S5). The total variance in the ARGs, explained by the selected variables, was 97.4% and Firmicutes, Actinobacteria and fat were favorably associated with RDA1 (explaining 33.95% of the total variance) and the C2 camel milk sample. Proteobacteria and MGEs were well correlated with RDA2 (explaining 18.02% of the total variance) and the C1 camel milk sample. The C3 and C4 camel milk samples were positively correlated with unidentified bacteria and Bacteroidota (Figure S5). Above all, this suggested that the distribution of ARGs in camel milk was mainly influenced by the bacterial community.

Then, the specific relationship between bacterial communities and the ARG distribution was revealed using Procrustes analysis (Figure 4B). The M^2^ and *p* values were 2.1225 and 0.032 in the Procrustes analysis, respectively, confirming that the composition of bacterial communities and ARGs in the camel milk samples was strongly associated (Figure 4B). Hence, bacterial community composition was the main factor shaping the ARG profiles in the camel milk samples, which was consistent with previous studies based on composting and pig manure [37,38]. Moreover, they are also often the main carriers of MGEs [39]. There is a potential risk of spreading ARGs through horizontal and vertical gene transfer (VGT and HGT).

### 3.5. Transmission of ARGs in Camel Milk

**Figure 5:**
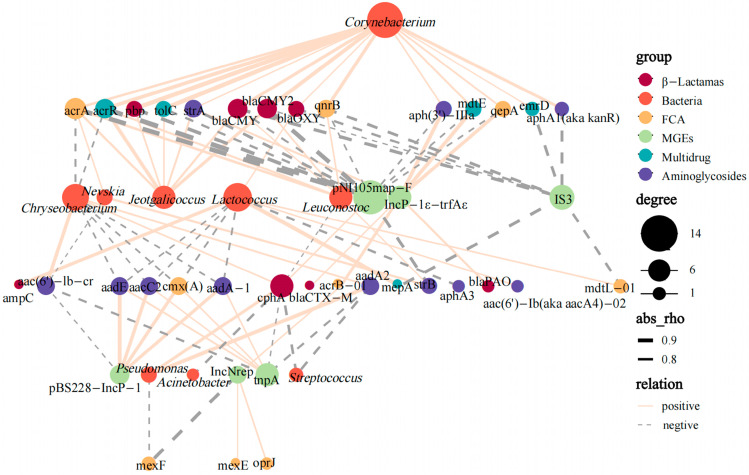
Network analysis reveals the genus-level co–occurrence pattern of bacterial taxa (16S rRNA gene sequence data), ARGs (β–lactamas, multidrugs, FCA and aminoglycosides) and MGEs (plasmid and transposon). The points are marked in colors corresponding to the different types of resistance genes, MGEs and genera, and the size represents the number of relevant objects. The solid and dashed lines indicate positive and negative correlations, respectively, with wider lines indicating stronger correlations (Spearman’s correlation, *p* < 0.05).

Previous studies have documented that ARGs can transfer among bacteria [40,41]. In this study, bacteria communities were found to play a vital role of shaping ARGs in camel milk. To determine the transmission of camel milk ARGs, network analysis (Figure 5) was used to visualize the co–occurrence of relationships between bacteria communities, MGEs and ARGs among samples. A total of 9 ARGs conferred resistance to aminoglycosides, β–lactamas and FCA in camel milk that displayed a high co–occurrence with MGEs. For example, pBS228–IncP–1α and tnpA (plasmid and transposon, respectively) were significantly correlated with cmx(A), aacC2, aadA–1 and aadE (r > 0.7, *p* < 0.01), IncNrep (plasmid) was shown to correlate strongly with acrB–01, mexE, and oprJ (r > 0.7, *p* < 0.01). IncP–1ε–trfAε (plasmid) also showed strong correlation with blaCTX–M and blaPAO (*p* < 0.01) (Figure 5). These suggest that HGT may play a vital role in the spread of aminoglycoside–, β–lactamas– and FCA–resistant genes in camel milk samples. More and more studies have reported that HGT is an important pathway for the transmission of ARGs, causing antibiotic-resistant bacteria to emerge in a variety of environments, as well as in raw milk [42,43].

However, rather than MGEs, more ARG subtypes in the camel milk samples displayed a high co–occurrence with bacterial communities (Figure 5). *Corynebacterium* were significantly correlated with blaCMY, blaCMY2, pbp, qnrB, acrR, tolC and aac–(6′)–lb–cr (r > 0.85, *p* < 0.001), similarly to *Leuconostoc* with blaCMY2, acrA and acrR (r > 0.75, *p* < 0.01) and *Lactococcus* with ampC and mepA (r > 0.85, *p* < 0.001) (Figure 5). These visualized linkages imply that VGT may play an essential role in the spread of ARGs in camel milk. It should be noted that *Leuconostoc* and *Lactococcus* showed correlation with β–lactamas–, multidrug–, and FCA–resistant genes in raw milk samples [44], and that the blaCMY gene could spreading across bacterial communities in raw milk [13]. Statistical correlation can be a valuable guide to identifying the primary transmission pathway, although it does not provide direct evidence of gene transfer [45]. Both vertical and horizontal transfer possibilities of different ARG subtypes in camel milk were investigated in the above study. Camel milk is directly related to the human diet. The complex nature of the production environment can cause rapid transmission of ARGs. Despite the fact that camel milk is subjected to thermal sterilization, it also poses huge risks to farmers or the spread of ARGs to the food chain [14]. From this perspective, it is necessary to develop control of the bacteria in camel milk and their potential impact on human health.

## 4. Conclusions

The presented results identify bacterial community composition and diversity of ARGs in raw Bactrian camel milk from Xinjiang. 16S rRNA gene sequencing indicated that *Acinetobacter* and *Pseudomonas* dominated camel milk with lower levels of *Lactococcus* and *Leuconostoc*. A total of 153 ARGs and MGEs were identified, which predominantly encode resistance to β–lactamas, multidrugs and FCA. Moreover, variation partitioning analysis (VPA), redundancy analysis (RDA) and Procrustes analysis implied that the bacterial community of the camel milk played a key factor in shaping the ARG profile. Meanwhile, network analysis even revealed a high degree of co–occurrence of ARGs with *Corynebacterium*, *Leuconostoc* and *Lactococcus*. In addition, ARGs also strongly correlated with pBS228–IncP–1α, IncP–1ε–trfAε and tnpA. So, the variety of ARGs in raw Bactrian camel milk is constantly spreading through VGT and HGT, and poses big risks to the food chain or, potentially, a risk to public health.

## Figures and Tables

**Table 1:** Chemical index composition of camel milk.

Farms	Protein (%)	Fat (%)	Nonfat Milk Solid (%)	Lactose (%)
C1	3.82 ± 0.03	4.19 ± 0.03	9.61 ± 0.03	5.07 ± 0.09
C2	3.79 ± 0.02	5.29 ± 0.18	9.48 ± 0.01	4.98 ± 0.02
C3	3.85 ± 0.07	4.74 ± 0.16	9.66 ± 0.02	5.08 ± 0.07
C4	3.82 ± 0.03	4.52 ± 0.07	9.64 ± 0.01	5.09 ± 0.02

Each value is presented as the mean ± standard error.

## Data Availability

All data needed to evaluate the conclusions in the paper are present in the paper and/or the Supplementary Materials.

## Supplementary Materials

The following supporting information can be downloaded at https://www.mdpi.com/article/10.3390/foods12213928/s1. Figure S1: the alpha diversity of bacterial communities in camel milk samples from four farms; Figure S2: principal component analysis (PCA) based on the unweighted Unifrac distance showing the bacterial communities’ compositions in camel milk from four farms; Figure S3: the number of ARGs detected in camel milk from four farms; Figure S4: Venn diagram of the number of ARGs detected in camel milk from four farms; Figure S5: redundancy analysis (RDA) to assess the relationship among physicochemical indicators (arrows), bacterial communities (arrows), mobile genetic elements (MGEs) and ARGs (symbols) in camel milk samples from four farms; Table S1: antibiotic resistance genes and mobile genetic elements; Table S2: alpha diversity analysis of bacterial communities in different farms; Table S3: Pearson correlation analysis between physicochemical indexes and absolute abundance of ARGs in camel milk; Table S4: correlations among absolute abundances of ARGs, MGEs and 16S rRNA.
