# Supplementary material for: The Antibiotic Resistome and Its Association with Bacterial Communities in Raw Camel Milk from Altay Xinjiang
Source: Foods. 2023 Oct 26;12(21):3928. doi: 10.3390/foods12213928 (PMC10647823; doi:10.3390/foods12213928)
Supplement: Supplementary file 1 [file foods-12-03928-s001.zip › foods-2662855-supplementary.pdf]

# Supporting Information

**Table S1.** Antibiotic resistance genes and mobile genetic elements.

| Number | Gene Name | ARDB Gene | Classification |
|---|---|---|---|
| 1. | 16S rRNA | 0 | 0 |
| 2. | aac | aac2ic | Aminoglycosides |
| 3. | aac(6')I1 | aac6ia | Aminoglycosides |
| 4. | aac(6')−Ib(aka aacA4)−01 | aac6ib | Aminoglycosides |
| 5. | aac(6')−Ib(aka aacA4)−02 | aac6ib | Aminoglycosides |
| 6. | aac(6')−Ib(aka aacA4)−03 | aac6ib | Aminoglycosides |
| 7. | aac(6')−II | aac6iia | Aminoglycosides |
| 8. | aac(6')−Iy | aac6iF | Aminoglycosides |
| 9. | aacA/aphD | aac6ie | Aminoglycosides |
| 10. | aacC | aac3vi | Aminoglycosides |
| 11. | aacC1 | aac3ia | Aminoglycosides |
| 12. | aacC2 | aac3iia | Aminoglycosides |
| 13. | aacC4 | aac3iv | Aminoglycosides |
| 14. | aadA−01 | ant3ia | Aminoglycosides |
| 15. | aadA−02 | ant3ia | Aminoglycosides |
| 16. | aadA1 | ant3ia | Aminoglycosides |
| 17. | aadA−1−01 | aph3iiia | Aminoglycosides |
| 18. | aadA−1−02 | aph3iiia | Aminoglycosides |
| 19. | aadA2−01 | ant2ia | Aminoglycosides |
| 20. | aadA2−02 | ant2ia | Aminoglycosides |
| 21. | aadA2−03 | ant3ia | Aminoglycosides |
| 22. | aadA5−01 | aadA5 | Aminoglycosides |
| 23. | aadA5−02 | aadA5 | Aminoglycosides |
| 24. | aadA9−01 | aad9 | Aminoglycosides |
| 25. | aadA9−02 | aad9 | Aminoglycosides |
| 26. | aadD | aadd | Aminoglycosides |
| 27. | aadE | ant6ia | Aminoglycosides |
| 28. | aph | spec−aph | Aminoglycosides |
| 29. | aph(2')−Id−01 | aph2 | Aminoglycosides |
| 30. | aph(2')−Id−02 | aph2 | Aminoglycosides |
| 31. | aph6ia | aph6ia | Aminoglycosides |
| 32. | aphA1(aka kanR) | aph3ia | Aminoglycosides |
| 33. | spcN−01 | spcN | Aminoglycosides |

| | | | |
|---|---|---|---|
| 34. | spcN−02 | spcN | Aminoglycosides |
| 35. | str | aph33ib | Aminoglycosides |
| 36. | strA | aph33ib | Aminoglycosides |
| 37. | strB | aph6id | Aminoglycosides |
| 38. | aphA3−01 | aphA3 | Aminoglycosides |
| 39. | aphA3−02 | aphA3 | Aminoglycosides |
| 40. | aac−(6′)−lb−cr | aac−(6′)−lb−cr | Aminoglycosides |
| 41. | aph(3′)−IIIa | aph(3′)−IIIa | Aminoglycosides |
| 42. | aadA25 | aadA25 | Aminoglycosides |
| 43. | acrA−01 | acra | FCA |
| 44. | acrA−02 | acra | FCA |
| 45. | acrA−03 | acrA | FCA |
| 46. | acrA−04 | acra | FCA |
| 47. | acrA−05 | acrA | FCA |
| 48. | acrB−01 | acrb | FCA |
| 49. | acrF | acrf | FCA |
| 50. | adeA | adea | FCA |
| 51. | catA1 | cata1 | FCA |
| 52. | catB3 | catb3 | FCA |
| 53. | cfr | cfr | FCA |
| 54. | cmeA | cmea | FCA |
| 55. | cmlA1−01 | cml_e1 | FCA |
| 56. | cmlA1−02 | cml_e1 | FCA |
| 57. | cmx(A) | cmx(A) | FCA |
| 58. | floR | cml_e3 | FCA |
| 59. | mexA | mexA | FCA |
| 60. | mexD | mexD | FCA |
| 61. | mexE | mexe | FCA |
| 62. | mexF | mexf | FCA |
| 63. | oprJ | oprj | FCA |
| 64. | pmrA | pmrA/MdtA | FCA |
| 65. | qnrA | qnra | FCA |
| 66. | yidY/mdtL−01 | yidy/mdtl | FCA |
| 67. | yidY/mdtL−02 | yidy/mdtl | FCA |
| 68. | qnrB | qnrB | FCA |
| 69. | qepA | qepA | FCA |
| 70. | bacA−01 | bacA | other |
| 71. | bacA−02 | baca | other |
| 72. | catB8 | catb8 | other |
| 73. | ceoA | ceoA | other |
| 74. | cmr | cmr | other |
| 75. | ereB | ereB | other |
| 76. | fabK | fabK | other |
| 77. | fosB | fosB | other |

| | | | |
|---|---|---|---|
| 78. | fosX | fosx | other |
| 79. | imiR | imiR | other |
| 80. | IS613 | transposase | other |
| 81. | marR−01 | marR | other |
| 82. | mdetl1 | mdetl1 | other |
| 83. | nimE | nimE | other |
| 84. | nisB | nisB | other |
| 85. | pncA | pncA | other |
| 86. | putitive multidrug | putitive multidrug | other |
| 87. | qac | qac | other |
| 88. | qacA | qaca | other |
| 89. | qacA/qacB | qaca/qacb | other |
| 90. | qacEdelta1−01 | qacEdelta1 | other |
| 91. | qacEdelta1−02 | qacEdelta1 | other |
| 92. | rarD−02 | rarD | other |
| 93. | sat4 | sat | other |
| 94. | sdeB | sdeB | other |
| 95. | speA | speA | other |
| 96. | Tp614 | tp614 | other |
| 97. | ttgA | ttgA | other |
| 98. | ttgB | ttgB | other |
| 99. | yceE/mdtG−01 | yceE/mdtG | other |
| 100. | yceE/mdtG−02 | yceE/mdtG | other |
| 101. | yceL/mdtH−01 | yceL/mdtH | other |
| 102. | yceL/mdtH−02 | yceL/mdtH | other |
| 103. | yceL/mdtH−03 | yceL/mdtH | other |
| 104. | mcr1 | mcr1 | other |
| 105. | mcr2 | mcr2 | other |
| 106. | blaI | blaI | other |
| 107. | blaRI | blaRI | other |
| 108. | binL | binL | other |
| 109. | blaOXA−23−like | blaOXA−23−like | other |
| 110. | catB2 | catB2 | other |
| 111. | fosA3 | fosA3 | other |
| 112. | dfrA32 | dfrA32 | other |
| 113. | dfrA14 | dfrA14 | other |
| 114. | msr(E) | msr(E) | other |
| 115. | optrA | optrA | other |
| 116. | poxtA | poxtA | other |
| 117. | vanD | vanD | other |
| 118. | copB | copB | other |
| 119. | pcoB | pcoB | other |
| 120. | mdfA | mdfA | other |
| 121. | ampC/blaDHA | bl1_ampc/dha | Beta_Lactamas |

| | | | |
|---|---|---|---|
| 122. | ampC−01 | bl1_ampC | Beta_Lactamas |
| 123. | ampC−02 | bl1_ec | Beta_Lactamas |
| 124. | ampC−04 | bl1_ec(ampC) | Beta_Lactamas |
| 125. | ampC−05 | bl1_ampc | Beta_Lactamas |
| 126. | ampC−06 | bl1_ampc | Beta_Lactamas |
| 127. | ampC−07 | bl1_ampc | Beta_Lactamas |
| 128. | ampC−09 | bl1_sm | Beta_Lactamas |
| 129. | bla1 | bl2a_iii | Beta_Lactamas |
| 130. | bla−ACC−1 | bl1_acc | Beta_Lactamas |
| 131. | blaCMY | bl1_cmy2 | Beta_Lactamas |
| 132. | blaCMY2−01 | bl1_cmy2 | Beta_Lactamas |
| 133. | blaCMY2−02 | bl1_cmy2 | Beta_Lactamas |
| 134. | blaCTX−M−01 | bl2be_ctxm | Beta_Lactamas |
| 135. | blaCTX−M−02 | bl2be_ctxm | Beta_Lactamas |
| 136. | blaCTX−M−03 | bl2be_ctxm | Beta_Lactamas |
| 137. | blaCTX−M−04 | bl2be_ctxm | Beta_Lactamas |
| 138. | blaCTX−M−05 | bl2be_ctxm | Beta_Lactamas |
| 139. | blaCTX−M−06 | bl2be_ctxm | Beta_Lactamas |
| 140. | blaGES | bl2_ges | Beta_Lactamas |
| 141. | blaIMP−01 | bl3_imp | Beta_Lactamas |
| 142. | blaIMP−02 | bl3_imp | Beta_Lactamas |
| 143. | bla−L1 | bl3_l | Beta_Lactamas |
| 144. | blaMOX/blaCMY | bl1_mox(cmy) | Beta_Lactamas |
| 145. | blaOCH | bl2a_okp | Beta_Lactamas |
| 146. | blaOKP | bl2d_oxa1/bl2d_oxa30 | Beta_Lactamas |
| 147. | blaOXA1/blaOXA30 | bl2d_oxa10 | Beta_Lactamas |
| 148. | blaOXA10−01 | bl2d_oxa10 | Beta_Lactamas |
| 149. | blaOXA10−02 | bl2be_oxy1 | Beta_Lactamas |
| 150. | blaOXY | bl1_pao | Beta_Lactamas |
| 151. | blaPAO | bl2be_per | Beta_Lactamas |
| 152. | blaPER | bl2c_pse1 | Beta_Lactamas |
| 153. | blaPSE | bl2c_pse1 | Beta_Lactamas |
| 154. | blaROB | bl2b_rob | Beta_Lactamas |
| 155. | blaSFO | blaSFO | Beta_Lactamas |
| 156. | blaSHV−01 | bl2be_shv2/2bl2_len | Beta_Lactamas |
| 157. | blaSHV−02 | bl2be_shv2 | Beta_Lactamas |
| 158. | blaTEM | bl2b_tem1 | Beta_Lactamas |
| 159. | blaTLA | blaTLA | Beta_Lactamas |
| 160. | blaVEB | bl2_veb | Beta_Lactamas |
| 161. | blaVIM | bl3_vim | Beta_Lactamas |
| 162. | blaZ | bl2a_pc | Beta_Lactamas |
| 163. | cepA | bl2e_cepa | Beta_Lactamas |
| 164. | cfiA | cfiA | Beta_Lactamas |
| 165. | cfxA | bl2e_cfxa | Beta_Lactamas |

| | | | |
|---|---|---|---|
| 166. | cphA−01 | bl3_cpha | Beta_Lactamas |
| 167. | cphA−02 | bl3_cpha | Beta_Lactamas |
| 168. | fox5 | fox5 | Beta_Lactamas |
| 169. | mecA | meca | Beta_Lactamas |
| 170. | ndm−1 | 0 | Beta_Lactamas |
| 171. | pbp | pbp | Beta_Lactamas |
| 172. | pbp2x | pbpp2x | Beta_Lactamas |
| 173. | Pbp5 | Pbp5 | Beta_Lactamas |
| 174. | penA | pbp2b/pena | Beta_Lactamas |
| 175. | carB | carB | MLSB |
| 176. | ereA | erea | MLSB |
| 177. | erm(34) | erm34 | MLSB |
| 178. | erm(35) | erm35 | MLSB |
| 179. | erm(36) | erm36 | MLSB |
| 180. | ermA | erma | MLSB |
| 181. | ermA/ermTR | erma/ermTR | MLSB |
| 182. | ermB | ermb | MLSB |
| 183. | ermC | ermc | MLSB |
| 184. | ermF | ermf | MLSB |
| 185. | ermJ/ermD | ermJ/ermD | MLSB |
| 186. | ermK−01 | ermK | MLSB |
| 187. | ermK−02 | ermK | MLSB |
| 188. | ermT−01 | ermt | MLSB |
| 189. | ermT−02 | ermt | MLSB |
| 190. | ermX | ermx | MLSB |
| 191. | ermY | ermy | MLSB |
| 192. | lmrA−01 | lmra | MLSB |
| 193. | lnuA−01 | lnua | MLSB |
| 194. | lnuB−01 | lnub | MLSB |
| 195. | lnuB−02 | lnub | MLSB |
| 196. | lnuC | lnuC | MLSB |
| 197. | matA/mel | matA/mel | MLSB |
| 198. | mdtA | mdtA | MLSB |
| 199. | mefA | mefa | MLSB |
| 200. | mphA−01 | mphA | MLSB |
| 201. | mphA−02 | mphA | MLSB |
| 202. | mphB | mphb | MLSB |
| 203. | mphC | mphc | MLSB |
| 204. | msrA−01 | msra | MLSB |
| 205. | msrC−01 | msrC | MLSB |
| 206. | oleC | oleC | MLSB |
| 207. | pikR1 | pikR1 | MLSB |
| 208. | pikR2 | pikR2 | MLSB |
| 209. | vatB−01 | vatB | MLSB |

| | | | |
|---|---|---|---|
| 210. | vatB−02 | vatB | MLSB |
| 211. | vatC−01 | vatC | MLSB |
| 212. | vatC−02 | vatC | MLSB |
| 213. | vatE−01 | vate | MLSB |
| 214. | vatE−02 | vate | MLSB |
| 215. | vgaA−01 | vgaa | MLSB |
| 216. | vgaA−02 | vgaa | MLSB |
| 217. | vgb−01 | vgb | MLSB |
| 218. | vgbB−01 | vgaB | MLSB |
| 219. | vgbB−02 | vgaB | MLSB |
| 220. | vatD | vatD | MLSB |
| 221. | vgaB−01 | vgaB | MLSB |
| 222. | vgaB−02 | vgaB | MLSB |
| 223. | intI−1(clinic) | 0 | MGEs |
| 224. | cIntI−1(class1) | 0 | MGEs |
| 225. | tnpA−06 | tnpA | MGEs |
| 226. | traN | traN | MGEs |
| 227. | IncQoriT | IncQoriT | MGEs |
| 228. | IncNrep | IncNrep | MGEs |
| 229. | IncPoriT | IncPoriT | MGEs |
| 230. | IncWrepA | IncWrepA | MGEs |
| 231. | intl2−01 | intl2−01 | MGEs |
| 232. | intl2−02 | intl2−02 | MGEs |
| 233. | intl3−01 | intl3−01 | MGEs |
| 234. | intl3−02 | intl3−02 | MGEs |
| 235. | pAKD1−IncP−1β | pAKD1−IncP−1β | MGEs |
| 236. | pAMBL−1−F | pAMBL−1−F | MGEs |
| 237. | pBS228−IncP−1α | pBS228−IncP−1α | MGEs |
| 238. | pNI105−F | pNI105−F | MGEs |
| 239. | pNI105map−F | pNI105map−F | MGEs |
| 240. | IncP−1ε−trfAε | IncP−1ε | MGEs |
| 241. | ISCR1 | ISCR1 | MGEs |
| 242. | IS26 | IS26 | MGEs |
| 243. | IS3 | IS3 | MGEs |
| 244. | tnpA−01 | Tn21 | MGEs |
| 245. | tnpA−02 | Tn22 | MGEs |
| 246. | tnpA−03 | tnpA | MGEs |
| 247. | tnpA−04 | Tn25 | MGEs |
| 248. | tnpA−05 | Tn24 | MGEs |
| 249. | tnpA−07 | Tn23 | MGEs |
| 250. | dfrA1 | dfra1 | Sulfonamides |
| 251. | dfrA12 | dfra12 | Sulfonamides |
| 252. | folA | folA | Sulfonamides |
| 253. | sul1 | sul1 | Sulfonamides |

| | | | |
|---|---|---|---|
| 254. | sul2 | sul2 | Sulfonamides |
| 255. | sulA/folP−01 | sulA/folP | Sulfonamides |
| 256. | sulA/folP−03 | sulA/folP | Sulfonamides |
| 257. | tet(32) | tet32 | Tetracyclines |
| 258. | tet(34) | tet34 | Tetracyclines |
| 259. | tet(35) | tet35 | Tetracyclines |
| 260. | tet(36)−01 | tet36 | Tetracyclines |
| 261. | tet(36)−02 | tet36 | Tetracyclines |
| 262. | tet(37) | tet37 | Tetracyclines |
| 263. | tetA−01 | teta | Tetracyclines |
| 264. | tetA−02 | teta | Tetracyclines |
| 265. | tetB−01 | tetb | Tetracyclines |
| 266. | tetB−02 | tetb | Tetracyclines |
| 267. | tetC−01 | tetC | Tetracyclines |
| 268. | tetC−02 | tetC | Tetracyclines |
| 269. | tetD−01 | tetd | Tetracyclines |
| 270. | tetD−02 | tetd | Tetracyclines |
| 271. | tetE | tete | Tetracyclines |
| 272. | tetG−01 | tetg | Tetracyclines |
| 273. | tetG−02 | tetg | Tetracyclines |
| 274. | tetH | teth | Tetracyclines |
| 275. | tetJ | tetj | Tetracyclines |
| 276. | tetK | tetk | Tetracyclines |
| 277. | tetL−01 | tetl | Tetracyclines |
| 278. | tetL−02 | tetl | Tetracyclines |
| 279. | tetM−01 | tetm | Tetracyclines |
| 280. | tetM−02 | tetm | Tetracyclines |
| 281. | tetO−01 | teto | Tetracyclines |
| 282. | tetPA | tetpa | Tetracyclines |
| 283. | tetPB−01 | tetpb | Tetracyclines |
| 284. | tetPB−02 | tetpb | Tetracyclines |
| 285. | tetPB−03 | tetpb | Tetracyclines |
| 286. | tetPB−04 | tetpb | Tetracyclines |
| 287. | tetPB−05 | tetpb | Tetracyclines |
| 288. | tetQ | tetq | Tetracyclines |
| 289. | tetR−02 | tetR | Tetracyclines |
| 290. | tetR−03 | tetR | Tetracyclines |
| 291. | tetS | tets | Tetracyclines |
| 292. | tetT | tett | Tetracyclines |
| 293. | tetU−01 | tetU | Tetracyclines |
| 294. | tetV | tetv | Tetracyclines |
| 295. | tetX | tetx | Tetracyclines |
| 296. | tet(38) | tet(38) | Tetracyclines |
| 297. | tetW−01 | tetW | Tetracyclines |

| | | | |
|---|---|---|---|
| 298. | vanA | vana | Vancomycin |
| 299. | vanB−01 | vanb | Vancomycin |
| 300. | vanB−02 | vanb | Vancomycin |
| 301. | vanC−01 | vanC | Vancomycin |
| 302. | vanC−03 | vanC | Vancomycin |
| 303. | vanC1 | vanC1 | Vancomycin |
| 304. | vanC2/vanC3 | vanC2/3 | Vancomycin |
| 305. | vanG | vang | Vancomycin |
| 306. | vanHB | vanhb | Vancomycin |
| 307. | vanHD | vanhd | Vancomycin |
| 308. | vanRA−01 | vanra | Vancomycin |
| 309. | vanRA−02 | vanra | Vancomycin |
| 310. | vanRB | vanrb | Vancomycin |
| 311. | vanRC | vanrc | Vancomycin |
| 312. | vanRC4 | vanrc | Vancomycin |
| 313. | vanRD | vanrd | Vancomycin |
| 314. | vanSA | vansa | Vancomycin |
| 315. | vanSB | vansb | Vancomycin |
| 316. | vanSC−02 | vansc | Vancomycin |
| 317. | vanSE | vanse | Vancomycin |
| 318. | vanTC−01 | vanTC | Vancomycin |
| 319. | vanTC−02 | vantc | Vancomycin |
| 320. | vanTE | vante | Vancomycin |
| 321. | vanTG | vantg | Vancomycin |
| 322. | vanWB | vanwb | Vancomycin |
| 323. | vanWG | vanwg | Vancomycin |
| 324. | vanXA | vanxa | Vancomycin |
| 325. | vanXB | vanxb | Vancomycin |
| 326. | vanXD | vanxd | Vancomycin |
| 327. | vanYB | vanyb | Vancomycin |
| 328. | vanYD−01 | vanyd | Vancomycin |
| 329. | vanYD−02 | vanyd | Vancomycin |
| 330. | acrR−01 | acrR | Multidrug |
| 331. | acrR−02 | acrR | Multidrug |
| 332. | emrD | emrd | Multidrug |
| 333. | mdtE/yhiU | mdtE/yhiU | Multidrug |
| 334. | mepA | mepA | Multidrug |
| 335. | mtrC−01 | mtrC | Multidrug |
| 336. | mtrC−02 | mtrC | Multidrug |
| 337. | mtrD−02 | mtrD | Multidrug |
| 338. | mtrD−03 | mtrD | Multidrug |
| 339. | oprD | oprd | Multidrug |
| 340. | qacH−01 | qacH | Multidrug |
| 341. | qacH−02 | qacH | Multidrug |

| | | | |
|---|---|---|---|
| 342. | tolC−01 | tolc | Multidrug |
| 343. | tolC−02 | tolc | Multidrug |
| 344. | tolC−03 | tolc | Multidrug |

**Table S2.** Alpha diversity analysis of bacterial communities in different farms.

| Sample Name | Shannon | Simpson | Chao1 | ACE |
|---|---|---|---|---|
| C1 | 4.44±0.59 | 0.84±0.05 | 1260.25±263.12 | 1352.62±265.46 |
| C2 | 4.75±0.55 | 0.88±0.03 | 1464.69±305.68 | 1539.17±291.85 |
| C3 | 5.59±1.56 | 0.90±0.06 | 1595.06±660.90 | 1657.44±674.41 |
| C4 | 4.26±0.35 | 0.79±0.01 | 1442.57±325.44 | 1502.25±328.62 |

Each value is presented as the mean ± standard deviation.

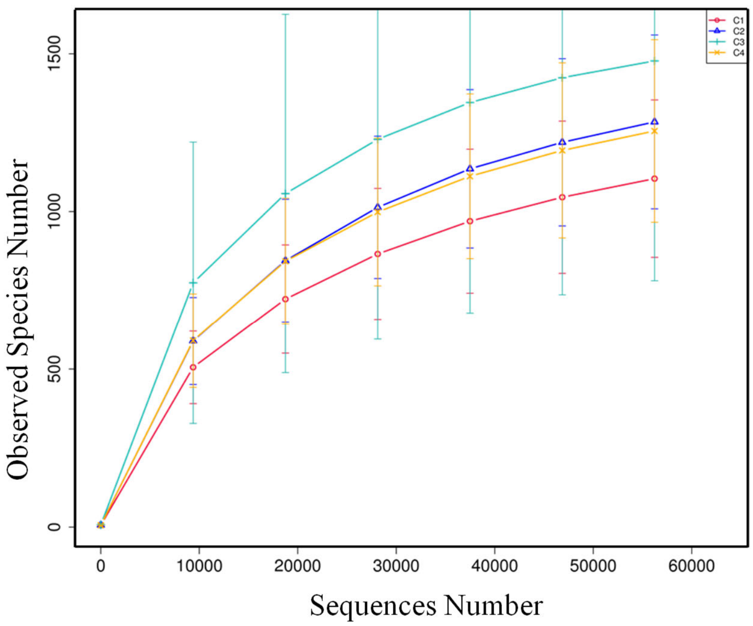


**Figure S1.** The alpha−diversity of bacterial community of camel milk samples from four farms.

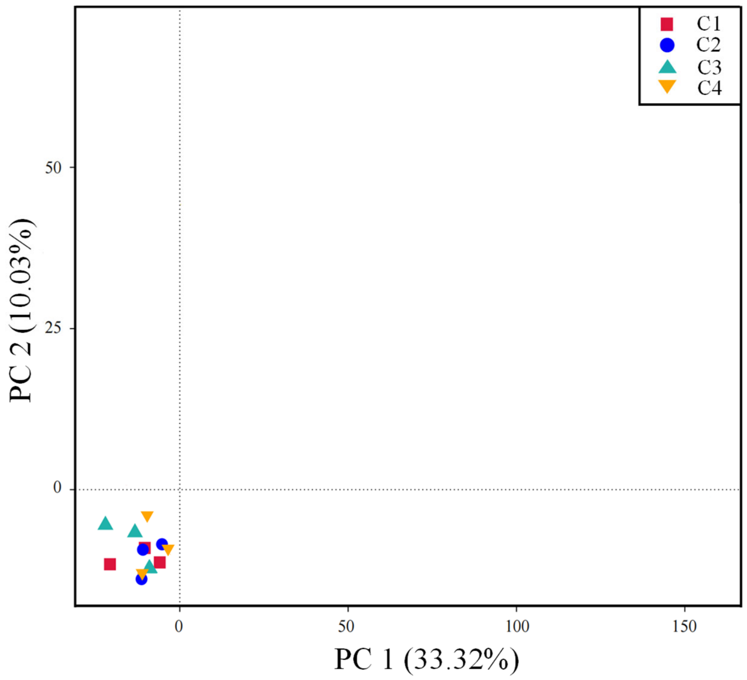


**Figure S2.** Principal component analysis (PCA) based on the Unweighted Unifrac distance showing the bacterial communities composition in camel milk from four farms.

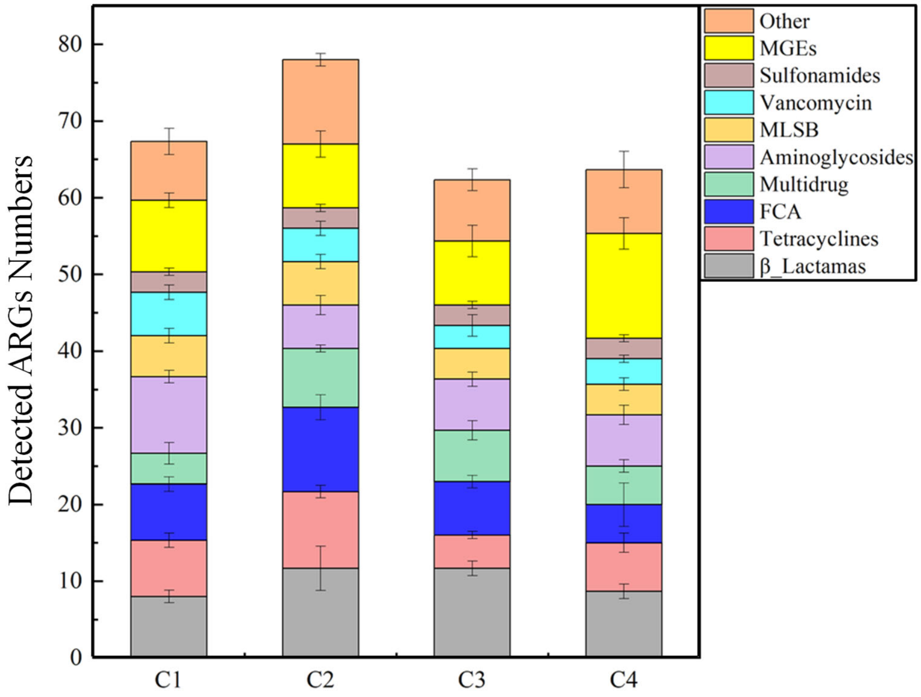


**Figure S3.** Number of ARGs detected in camel milk from four farms.

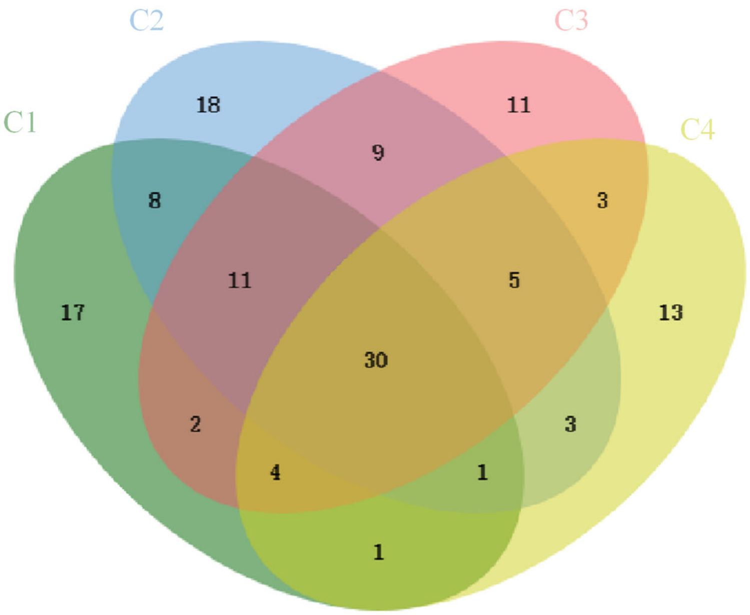


**Figure S4.** Venn diagram of the number of ARGs detected in camel milk from four farms.

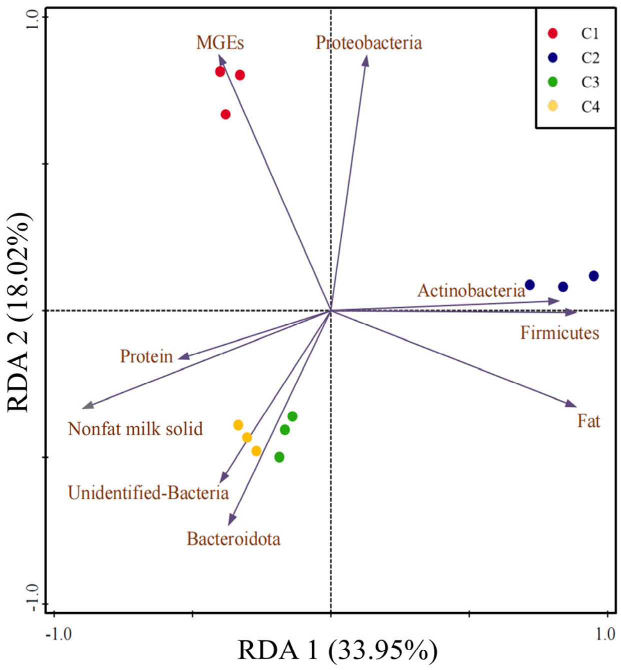


**Figure S5.** Redundancy analysis (RDA) assess the relationship among physicochemical indicators (arrows), bacterial communities (arrows), mobile genetic elements (MGEs), and ARGs (symbols) in camel milk samples from four farms.

**Table S3.** Pearson correlation analysis between physicochemical indexes and absolute abundance of ARGs in camel milk.

| | **Protein** | | **Fat** | | **Not−fat milk solid** | | **Lactose** | | **Ash** | |
|---|---|---|---|---|---|---|---|---|---|---|
| | **r** | **p** | **r** | **p** | **r** | **p** | **r** | **p** | **r** | **p** |
| Aminoglycosides RGs | −0.50 | 0.09 | 0.49 | 0.10 | −0.92*** | 0.00 | −0.62* | 0.02 | −0.28 | 0.36 |
| FCA RGs | −0.59* | 0.04 | 0.84*** | 0.00 | −0.93*** | 0.00 | −0.64* | 0.02 | −0.23 | 0.46 |
| Multidrug RGs | −0.57 | 0.05 | 0.83*** | 0.00 | −0.93*** | 0.00 | −0.66* | 0.01 | −0.24 | 0.45 |
| β−lactamas RGs | −0.57* | 0.04 | 0.83*** | 0.00 | −0.93*** | 0.00 | −0.66* | 0.01 | −0.24 | 0.43 |
| MLSB RGs | 0.14 | 0.66 | −0.53 | 0.07 | 0.09 | 0.77 | −0.08 | 0.79 | −0.18 | 0.58 |
| Sulfonamides RGs | −0.58* | 0.04 | 0.83*** | 0.00 | −0.93*** | 0.00 | −0.66* | 0.01 | −0.25 | 0.43 |
| Tetracyclines RGs | −0.10 | 0.75 | −0.59* | 0.04 | −0.10 | 0.74 | 0.02 | 0.94 | −0.10 | 0.74 |
| Vancomycin RGs | −0.14 | 0.66 | −0.68* | 0.01 | 0.02 | 0.95 | 0.10 | 0.74 | −0.19 | 0.53 |
| Other RGs | −0.61* | 0.03 | 0.85*** | 0.00 | −0.90*** | 0.00 | −0.64 | 0.02 | −0.30 | 0.34 |
| Total ARGs | −0.58* | 0.04 | 0.82** | 0.00 | −0.94*** | 0.00 | −0.66* | 0.01 | −0.24 | 0.43 |
| Total MGEs | 0.05 | 0.86 | −0.66* | 0.01 | 0.09 | 0.76 | 0.24 | 0.43 | 0.02 | 0.94 |
| No. ARGs | −0.55 | 0.06 | 0.46 | 0.14 | −0.84*** | 0.00 | −0.52 | 0.08 | −0.38 | 0.23 |
| 16S rRNA | 0.25 | 0.44 | 0.39 | 0.20 | −0.70* | 0.01 | −0.58* | 0.04 | −0.26 | 0.42 |

***: P<0.001 (double−tailed); **: *P*<0.01 (double−tailed); *: *P*<0.05 (double−tailed).

**Table S4**. Correlations among absolute abundances of ARGs, MGEs, and 16S rRNA.

| | | **Integron** | **Tranposon** | **Total MGEs** | **16S rRNA** |
|---|---|---|---|---|---|
| Aminoglycosides | r | −0.77** | 0.08 | 0.08 | 0.84*** |
| RGs | p | 0.00 | 0.80 | 0.80 | 0.00 |
| FCA RGs | r | −0.96*** | 0.08 | −0.31 | 0.65* |
| | p | 0.00 | 0.80 | 0.32 | 0.02 |
| Multidrug RGs | r | −0.97*** | −0.28 | −0.28 | 0.69* |
| | p | 0.00 | 0.37 | 0.37 | 0.01 |
| β−lactamas RGs | r | −0.97*** | −0.32 | −0.32 | 0.69* |
| | p | 0.00 | 0.31 | 0.31 | 0.01 |
| MLSB RGs | r | 0.31 | 0.86*** | 0.86*** | 0.38 |
| | p | 0.32 | 0.00 | 0.00 | 0.22 |
| Sulfonamides | r | −0.97*** | −0.32 | −0.32 | 0.69* |
| RGs | p | 0.00 | 0.30 | 0.30 | 0.01 |
| Tetracyclines | r | 0.32 | 0.92*** | 0.92*** | 0.40 |
| RGs | p | 0.30 | 0.00 | 0.00 | 0.19 |
| Vancomycin | r | 0.42 | 0.94*** | 0.94*** | 0.23 |
| RGs | p | 0.17 | 0.00 | 0.00 | 0.47 |
| Other RGs | r | −0.94*** | −0.42 | −0.42 | 0.56 |
| | p | 0.00 | 0.17 | 0.17 | 0.05 |
| Total ARGs | r | −0.97*** | −0.28 | −0.28 | 0.70* |
| | p | 0.00 | 0.38 | 0.38 | 0.01 |

***: P<0.001 (double−tailed); **: *P*<0.01 (double−tailed); *: *P*<0.05 (double−tailed).
